# BRCA germline mutation test for all woman with ovarian cancer?

**DOI:** 10.1186/s12885-019-5829-4

**Published:** 2019-06-28

**Authors:** A. V. Paradiso, M. Digennaro, M. Patruno, S. De Summa, S. Tommasi, I. Berindan-Neagoe

**Affiliations:** 1 Experimental Oncology - Center for Study of Heredo-Familial Tumors, IRCCS-Istituto Tumori “Giovanni Paolo II”, viale Orazio Flacco, 65, 70124 Bari, Italy; 2 Molecular and Pharmacogenetics Diagnostic Laboratory, IRCCS-Istituto Tumori “Giovanni Paolo II”, viale Orazio Flacco, 65, 70124 Bari, Italy; 3 Department of Functional Genomics and Experimental Pathology, The Oncology Institute “Prof. Dr. Ion Chiricuta”, University of Medicine and Pharmacy Iuliu Hatieganu- Center for Functional Genomics and Center for Advanced Medicine Medfuture, Republicii 34th street; Marinescu 23, Pasteur 4-6, 400015 Cluj-Napoca, Romania; 4 Centro Studi Tumori Eredo-Familiari, Istituto Tumori G Paolo II IRCCS National Cancer Research Centre, Via O Flacco, 65, 70124 Bari, Italy

**Keywords:** Ovarian cancer, BRCA, Test criteria

## Abstract

**Background:**

Delivering widespread BRCA testing to patients with ovarian cancer has been suggested by several scientists, recommended by professional societies and solicited by patients organizations. However, based on the lack of studies clearly demonstrating the cost-effectiveness of such approach compared to standard practice, we evaluated the possibility to better select subgroups of ovarian cancer (OC) patients with higher probability to be a BRCA mutation carrier’.

**Methods:**

We analyzed the database of 2222 germline BRCA analyses from OC patients recently published by Song et al. (Song 2014) by applying multivariate and conditional inference regression tree-analyses.

**Results:**

Overall, 178/2192 (8.1%) evaluable OC women showed pathogenic germline mutations in BRCA genes (84 BRCA1;94 BRCA2). BRCA mutations resulted significantly more frequent in Epithelial tumors (10.7%), less differentiated tumours (11.0%) and younger subjects (13.4%). Regression tree analysis permitted to individualize a subset of 66% OC patients with particularly low risk (3.5%) to carry a BRCA mutation vs a subgroup (24% of the series), with a probability higher than 17% to carry a pathogenic mutation. Younger age, OC and Breast Cancer family history were confirmed powerful factors in selecting subgroups of patients with significantly different BRCA mutation probability.

**Conclusions:**

Our regression tree-analysis can represent an innovative approach taking into consideration all main clinical pathological information to select OC patients to be candidated for BRCA test.

## Background

BRCA 1/2 genes play a major role in normal cell DNA repair machinery, participating in the repair of double-strand breaks by homologous recombination, which, when impaired, is responsible for accumulation of genomic alterations and final genomic instability [1].

The knowledge of these mechanisms permitted to interpret the pathogenic relevance of BRCA1/2 gene mutations in families carrying germline mutations: alterations in these genes confer an higher risk for ovarian-fallopian cancer ranging between 39 and 63% for BRCA 1 and 16–27% for BRCA2 mutations [2] with respect to a mean risk in the overall population of 1–2%. As a consequence, clinical preventive-prophylactic strategies have been proposed (www.nccn.org) for subjects carrying pathogenic mutations in these genes thus making urgent the individualization of appropriate criteria to candidate woman to BRCA1/2 genetic test [3]. The problem of best criteria for genetic counseling enrollment was debated since the last decade generally concluding that presence of familiarity and high risk for BRCA mutation probability calculated by specific softwares [4] should be utilized in routine clinical practice.

More recently, a new and exciting application for BRCA mutation test has been represented by its utilization as Companion Diagnostic Tests (CDX) for drugs Poly-ADP ribose polymerase-Inhibitors (PARP-I). PARP genes encode for proteins playing an essential role in DNA single-strand breaks repair suggesting that specific PARP-I drugs could be of clinical usefulness first of all by inducing a synthetic lethality effect in cells also carrying a BRCA gene mutations [5]. In 2014, Food and Drug Administration (www.FDA.gov) and European Medicines Agency (www.ema.europa.eu) confirmed the predictive value of BRCA test as CDX for selection of patients to PARP-I treatment after previous response to platinum agents [6, 7]. Considering that several studies reported that an important fraction (about 40%) of BRCA-mutated ovarian cancer (OC) patients might not have a family history [8] and, furthermore, that BRCA mutational status could provide information also regarding the prognosis and the therapeutic strategy overall, delivering widespread BRCA testing to all OC patients has been proposed by several Authors [8, 9], recommended by professional societies [10–12], requested by patients organizations [13, 14] and adopted by several authoritative institutions [15, 16].

The lack of studies clearly demonstrating the cost-effectiveness of widespread BRCA test approach with respect to nowadays standard practice, convinced us of the opportunity to verify if we can better select subgroups of OC patients to be tested for BRCA assay. In order to individualize subsets of cases with higher probability to carry a BRCA1/2 mutation, we applied for the first time multivariate and decision-tree analyses to the largest published database of OC patients, provided of exhaustive clinical and genetic associated information [17].

## Methods

### Clinical dataset

We analysed the database provided by CR-UK, Department of Oncology, University of Cambridge, UK, comprising clinical-pathological and molecular information of 2222 OC women with analyzed BRCA status. Data on germline BRCA analysis by Sanger-sequencing performed in all patients have been already published [17]. All patients included in the database had a previous histological diagnosis of invasive OC within two case-controls studies: the population-based SEARCH study (1321 cases) from the United Kingdom, and the Hospital-Mayo clinic study (919 cases) from USA [17].

For 2192 patients the following information were available: Age at diagnosis of OC; Ethnicity (only white woman); Histology (serous, mucinous, endometrioid, clear cell, mixed cell; other specified epithelial OC); cytohistological differentiation (Well, Moderately, Poorly or Undifferentiated Grade, Not assessed); disease stage according to FIGO classification; family history of OC in first degree relative (OCFh); family history of breast cancer in first degree relative (BCFh). The characteristics of the patient series are described in Table 1.

**Table 1:** Description of the clinical-pathological characteristics of the cohort of Ovarian Cancer patients **(**M&M for details on categories reported)

Histology	N (%)
Serous Epithelial	1312 (59,8)
Mucinous	143 (6,5)
Endometrioid	322 (14,7)
Clear Cell	201 (9,2)
Mixed	98 (4,5)
Other Epithelial (Brunner)	79 (3,6)
Undifferentiatied	5 (0,2)
N/A	32 (1,3)
Disease Stage FIGO	
1	523 (23,9)
2	249 (11,4)
3	1103 (50,3)
4	25 (1,1)
N/A	292 (13,3)
Grade Differentiation	
Well	447 (20,4)
Moderately	261 (11,9)
Poorly	1255 (57,2)
Undifferentiated	15 (0,7)
N/A	214 (9,8)
Ovarian Cancer family History	
No	1718 (78,4)
Yes	107 (4,9)
N/A	367 (16,7)
Breast Cancer Family History	
No	1519 (69,2)
Yes	319 (14,5)
N/A	354 (16,1)
BRCA1/2 alterations	
Present	182 (8,4)
Absent	2010 (91,6)

### Statistical methods

The frequencies of all clinical-pathological characteristics already described with respect to presence/absence of BRCA1/2 mutations were preliminarily analyzed by logistic regression; patients were grouped for further analysis according to histology of the tumour (epithelial vs not-epithelial histology), clinical disease stage (I-II vs III-IV) Differentiation grade (1–2 vs 3–4), Age (< 50 yrs. vs > 50 yrs), family history of OC (OCFh, present vs absent); family history of breast cancer (BCFh, present vs absent).

A multivariate logistic regression analysis with BRCA mutation status as dependent variable was conducted; all the variables included in the model were categorized as described above.

Finally, a statistical inference analysis was conducted building up a conditional inference tree. Conditional inference trees were performed with the “party” package (version 1.2–2) in the R system for statistical computing (version 3.3.2, R Development Core Team 2004), both being freely available [18] from CRAN (http://CRAN.R-project.org). In detail, for the present study, the tree function has been used to obtain conditional inference regression tree [19]. Such an approach integrates tree-structured regression models with conditional inference methods. Rpart algorithm in default mode, as used in the present paper, manages missing values keeping observations even if one or more predictors are lacking.

## Results

Overall, 178 (8.1%) of OC women showed a germline mutation in BRCA genes, 84 in BRCA 1 and 94 in BRCA2.

The frequency of BRCA mutations resulted higher in Epithelial tumors than in those of not epithelial origin (10.7% vs 4.2%; *p* < 0.0001); in less vs high differentiated tumours (11.0% vs 4.3%; p < 0.0001) and in younger < 50 years subjects (13.4% vs 6.8%; p < 0.0001). However, the highest probability to carry a BRCA mutation has been observed in cases with first degree family history of OC (27.1%) or BC (17.6%). Logistic regression analysis (Table 2), confirmed the significant difference between women with vs without OCFh (Odds ratio, OR: 4.9; 95% CI 3.04–7.73) and for women with vs without BCFh (OR: 3.19; 95% CI 2.22–4.53).

**Table 2:** Logistic Regression Analysis with BRCA mutation status as dependent variable in different clinical-pathological subsets of cases

Logistic Regression		
	Odds ratio (95% CI)	*p*-value
Histology		
Non Epithelial vs Epithelial	0.37 (0.25÷0.53)	< 0.0001
Stage		
II-III vs I	2.69 (1.7÷4.49)	< 0.0001
CytoHistological Differentiation Grade		
III vs I-II	2.7 (1.83÷4.1)	< 0.0001
Age		
> 50 years vs < 50 years	0.47 (0.34÷0.65)	< 0.0001
Ovarian Cancer history		
1st Degree Affected vs Not	4.9 (3.04÷7.73)	< 0.0001
Breast Cancer history		
1st Degree Affected vs Not	3.19 (2.22÷4.53)	< 0.0001

The multivariate regression analysis (Table 3), confirmed the predictive role for presence of BRCA mutation, of OCFh (OR 3.91;95%CI 2.1–7.04) and of BCFh (OR 3.75; 2.46–5.67); conversely, older> 50 yrs. women showed a significantly lower probability (OR 0.27; 95%CI: 0.18–0.43) to be BRCA1/2 mutation carriers.

**Table 3:** Multivariate Logistic Regression Analysis with BRCA mutation status as dependent variable in different clinical-pathological subsets of cases

Multivariate Logistic Regression		
	Odds ratio (95% CI)	*p*-value
Histology		
Non Epithelial vs Epithelial	0.48 (0.26÷0.83)	0.012
Stage		
II-III vs I	1.43 (0.76÷2.82)	0.28
CytoHistological Differentiation Grade		
III vs I-II	2.34 (1.34÷4.3)	0.003
Age		
> 50 years vs < 50 years	0.27 (0.18÷0.43)	< 0.0001
Ovarian Cancer history		
1st Degree Affected vs Not	3.91 (2.1÷7.04)	< 0.0001
Breast Cancer history		
1st Degree Affected vs Not	3.75 (2.46÷5.67)	< 0.0001

**Fig. 1:**
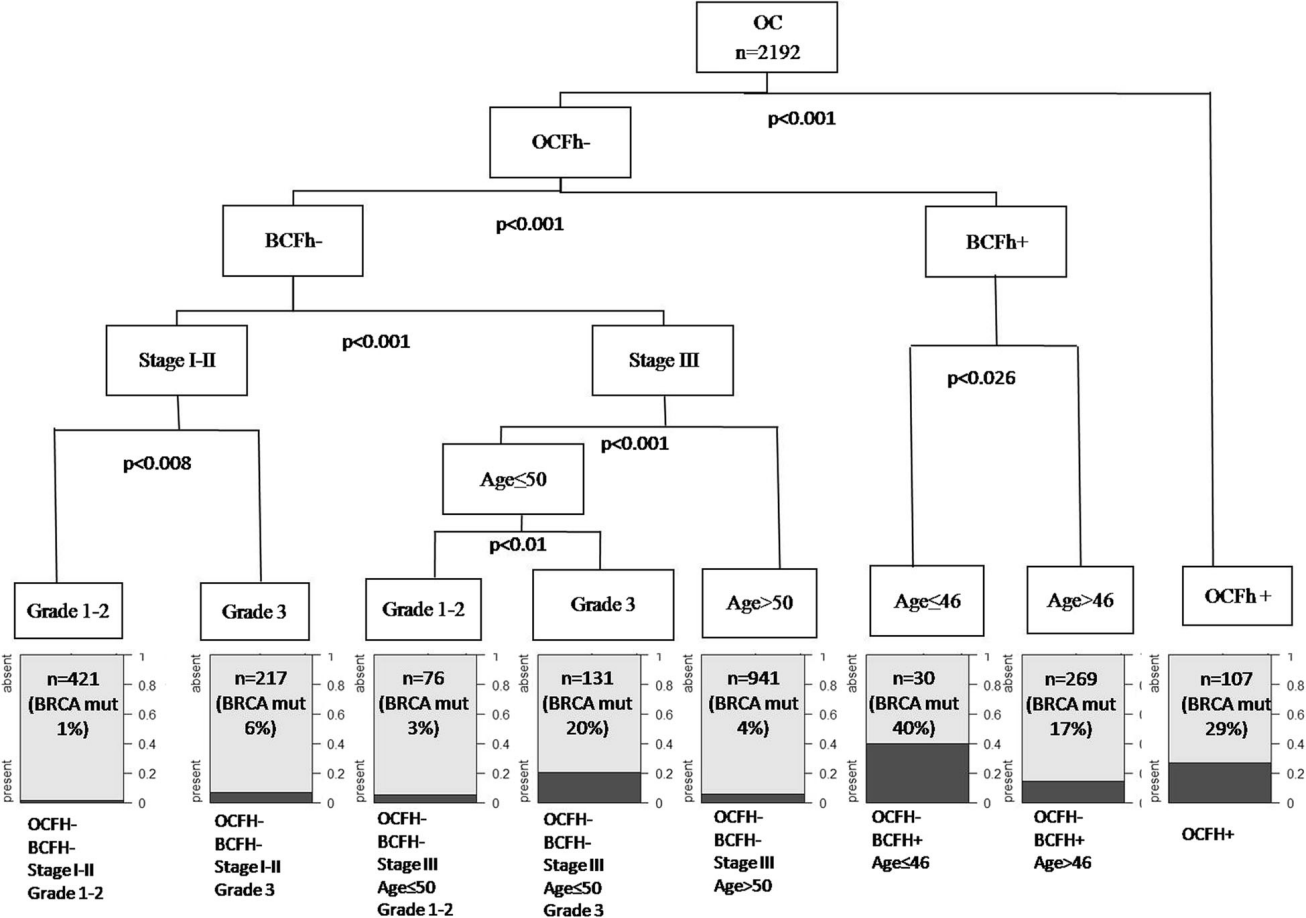
Regression tree for prediction of probability to find BRCA pathogenic mutation carriers in a series of 2192 ovarian cancer patients. Variable included in the model: Age, Family History (Fh) of Breast (BC) or Ovarian cancer (OC), Disease Stage, Cyto-Histological Grade. In leaves indicated number of cases and (in parenthesis) % of BRCA mutated carriers

In order to check for the possibility to better select subgroups of women with different probability frequency of BRCA mutations, we applied a regression tree analysis to a large series of clinical and molecular well characterized OC patients (Song, 2014). We highlighted a decisional tree with nodes indicating subgroups of patients significantly different for probability to carry a BRCA mutation (Fig. 1) with 8 terminal subgroups of OC women with a probability to carry a mutation ranging from 1 to 40%. This probability resulted particularly high (40%) in OC patients younger than 46 yrs., with BCFh and OCFh (NODE 6); in OC patients with OCFh+ (29%) (NODE 8). Conversely, that probability resulted: very limited (< 1% of BRCA mutation frequency) in patients with negative OCFh and BCFh/Disease Stage I-II/ Differentiation Grade I-II (NODE 1); in patients without OCFh and BCFh/Disease Stage III/ Younger Age/ Grade I-II (3% of overall series of patients with BRCA mutation frequency < 3%) (NODE 3).

## Discussion

Previous papers highlighted the great variability in terms of percentage of BRCA alteration in series of OC patients reported by different Authors. de-Jong [9] reported an overall probability of presence of germline BRCA1/2 mutations above 10% in 6218 women with epithelial OC confirming three referral criteria to candidate OC women to genetic counseling: age of onset, family history of BC and/or OC and histology. However, he also stressed that categories not fulfilling these selection criteria still have a substantial probability of carrying a germline BRCA mutation concluding that “testing should be offered regardless of those characteristics otherwise an important part of germline BRCA1/2 mutation carriers could be missed” [9]. This position was supported by several authors [8]. Furthermore, the recent updated 3.2019 NCCN guidelines do no longer consider the possibility to utilize genetic risk models (like BRCAPRO) for a better selection of candidates to BRCA test (https://www.nccn.org/professionals/physician_gls/pdf/genetics_screening.pdf).

In the present series we showed by univariate and multivariate analyses that BRCA mutation rate is strongly associated with epithelial histology, low citohistological tumour differentiation and, first of all, with OC or BC family history in first degree relatives (Tables 2 and 3). Several Authors confirmed that the epithelial cancer histology is associated to BRCA mutations. Indeed, Alsop [20] reported a germline BRCA1/2 mutation in 14% of 1001 women with non-mucinous epithelial ovarian cancer (EOC); Zhong [21] pointed out the presence of 17% of BRCA mutations by reviewing a series of 9588 epithelial EOC. In our series of patients, we demonstrated 10.7% percentage of BRCA mutation in epithelial origin OC women; however, as already reported [17], gene deletions and duplications in this series were not analyzed, even if, Kwong [22] demonstrated that large deletions or duplications in BRCA1/2 genes, accounts for 0.7% of all BRCA pathogenic alterations, only.

The most intriguing results of the present paper, came from regression tree analysis which showed that there are subgroups of patients, characterized by a combination of clinical-pathological factors and an enormous difference in BRCA mutation frequencies. For first our original analysis confirms the strong impact that OC and/or BC family history has in determining the probability to carry a BRCA mutation and this, irrespective to age for OC.

We further demonstrated that there is a subgroup of about 20% of all OC patients (without family history, early disease stage, well differentiated) with < 1% of BRCA mutation rate; moreover, a subgroup of 46% of patients, included in Nodes 3 and 5 of the Regression-Tree (Fig. 1) showed a < 3.8% probability only to carry a BRCA mutation. Conversely, this probability resulted particularly high (> 40%) in young women with BCFh (Node 6) or with OCFh (> 29%) (Node8). This is the first time that the concept of hierarchy and of multifactor risk is associated to BRCA mutation rate in a large series of OC women. In fact, our hierarchical approach permitted to individualize patients belonging to Nodes 1–3-5 of the Tree, representing a subset of 66% of all OC patients, with particularly low risk to carry a BRCA mutation vs a subgroup of OC women, representing the 24% of all the series, with a probability to carry a pathogenic mutation always over 17%.

The main conclusion from these data is that the probability to find a BRCA mutation varies greatly in different clinical subgroups leading to the hypothesis that testing for BRCA mutations in OC patients could be better addressed within each specific clinical scenario and according to better defined cost-effective programs.

The question of how to manage, in a cost-effective way, BRCA tests for OC patients has generated a wide debate. D’Andrea [23], after a systematic review on economic evaluations on BRCA genetic testing programs, concluded that there is no evidence of cost-effectiveness for BRCA screening to all newly diagnosed cases of OC cancer even though followed by cascade testing of relatives. Kwon [24], estimating the cost-effectiveness of BRCA mutation testing in USA and the down stream benefits for first degree relatives, confirmed that the benefit concerned only OC women, with a personal history of breast and/or OC. Slade [25] stressed the need for adherence to NICE elegibility criteria requiring a BRCAPRO risk> 10% to reach a useful cost-effectiveness ratio. Eccleston [26], in a study conducted in UK, reached different conclusions reporting that implementing routine BRCA testing in women with OC would be cost-effective but only if compared with no testing to all patients policy.

We can therefore affirm that there is no evidence of a clear cost-efficacy benefit for widespread genetic test to all OC patients when compared to testing selected subgroups of patients only. On the other hand, we have to stress that there is no demonstration that our regression tree model can represent an alternative more cost-effective approach with respect to standard practice. A study to directly compare the performances of our innovative approach with respect to BRCAPRO is ongoing.

However, there is general agreement about the fact that new technological approach (i.e. massive sequencing) will dramatically lower costs and then the cost-efficacy equilibrium for wide BRCA test utilization policies [22–24].

An important point supporting the implementation of widespread testing strategies has been the utilization of such test as predictive biomarker for PARP-I stated from FDA and EMA, and, more in general, for an optimal therapeutic strategy design for OC women [8, 27]. In particular, PARP-I utilized in OC women carrying a BRCA mutation as maintenance therapy, has proven to dramatically improve the outcome of these patients [28].

However, regarding these points, alternative views have to be discussed. In 2017, the FDA (www.accessdata.fda.gov) approved two PARP inhibitors, olaparib and niraparib, as maintenance treatment for women with OC who respond to induction platinum-based chemotherapy, regardless of their BRCA-mutation status [28, 29]; recent findings from the phase III ARIEL3 trial of rucaparib corroborate the genotype agnostic benefit of PARP inhibition [30]. Moreover, Tan [31] supported the idea that the delivery of BRCA test as predictive to response to other common drugs (platinum derivatives, trabectedin) utilized for OC patients has to be still considered as an experimental approach. It seems we can conclude that, to date, to know the BRCA test to consider PARP-I utilization and for a better planning a complete therapeutic strategy for OC patients, cannot be simply supported.

Interestingly, the ARIELIII trial scientists stressed the urgent need to a deeper study of homologous recombination repair deficiency (HRD) in patients candidate to PARP-I treatment, also considering the potential harmful effect of false negative BRCA results leading to false patient’s reassurance and to appropriate care neglection [30].

## Conclusions

In conclusion, while some countries are already considering national wide roadmaps to facilitate and improve BRCA genetic testing rates [32], we suggest that there is no evidence that delivering a widespread BRCA testing for OC patients is cost effective with respect to standard practice for preventive and therapeutic purposes. The possibility to better select candidates to the test is a feasible approach and, from this perspective, our regression tree analysis could represent a reasonable practical approach. A better selection of patients to be tested together with new predictive biomarkers looking, in a deeper and wider way, at HRD characteristics of OC patients are urgently needed.

## Data Availability

All data materials and analyses are available upon request and only after approval of UK-CRUK Referents

## Abbreviations

BC: Breast cancer
BCFh: Breast Cancer in first degree relative.
CDX: Companion Diagnostic Text
HRD: homologous recombination DNA repair deficiency
OC: Ovarian Cancer
OCFh: Ovarian Cancer in first degree relative
PARP-I: Poly-ADP ribose polymerase-Inhibitors
